# Arrhythmogenic left ventricular cardiomyopathy caused by a novel likely pathogenic *DSP* mutation, p.K1165Rfs*8, in a family with sudden cardiac death

**DOI:** 10.1186/s12920-023-01701-w

**Published:** 2023-10-26

**Authors:** Amir Azimi, Maryam Pourirahim, Golnaz Houshmand, Sara Adimi, Majid Maleki, Samira Kalayinia

**Affiliations:** 1 Rajaie Cardiovascular Medical and Research Center, Iran University of Medical Sciences, Tehran, Iran University of Medical Sciences, Tehran, Iran; 2 Cardiogenetic Research Center, Rajaie Cardiovascular Medical and Research Center, Iran University of Medical Sciences, Tehran, Iran

**Keywords:** Arrhythmogenic left ventricular cardiomyopathy, Genetic, Desmoplakin, Whole-exome sequencing, Variant

## Abstract

**Objective:**

We conducted an investigation into the clinical and molecular characteristics of Arrhythmogenic left ventricular cardiomyopathy (ALVC) caused by a novel likely pathogenic mutation in an Iranian pedigree with sudden cardiac death (SCD).

**Background:**

ALVC is a genetically inherited myocardial disease characterized by the substitution of fibro-fatty tissue in the left ventricular myocardium, predominantly inherited in an autosomal dominant pattern and is commonly associated with genes involved in encoding desmosomal proteins, specifically Desmoplakin (*DSP*).

**Methods:**

The patient and available family members underwent a comprehensive clinical assessment, including Cardiac magnetic resonance (CMR) imaging, along with Whole-exome sequencing (WES). The identified variant was confirmed and segregated by Polymerase chain reaction (PCR) and Sanger sequencing in the family members.

**Results:**

A novel likely pathogenic heterozygous variant, *DSP* (NM_004415.4), c.3492_3498del, p.K1165Rfs*8 was discovered in the proband. This variant is likely to be the primary reason for ALVC in this specific family. This variant was confirmed by Sanger sequencing and segregated in other affected members of the family.

**Conclusion:**

We identified a novel likely pathogenic variant in the *DSP* gene, which has been identified as the cause of ALVC in an Iranian family. Our investigation underscores the importance of genetic testing, specifically WES, for individuals suspected of ALVC and have a family history of SCD.

**Supplementary Information:**

The online version contains supplementary material available at 10.1186/s12920-023-01701-w.

## Introduction

Arrhythmogenic cardiomyopathy (ACM) is a genetically inherited myocardial disease characterized by the replacement of ventricular myocardium with fibro-fatty tissue. It is typically inherited in an autosomal dominant pattern, with prevalence estimates ranging from 0.02% to 0.1% of the population. ACM is related to ventricular arrhythmias, sudden cardiac death (SCD), and progressive heart failure, particularly in young males and athletes [1–4]. Arrhythmogenic left ventricular cardiomyopathy (ALVC), also known as the left-dominant type, is characterized by predominant left ventricular (LV) involvement with minor or no right-sided abnormalities [2, 3]. Clinical manifestations of ALVC include electrocardiogram (ECG) changes like low-amplitude QRS complexes in limb leads and T-wave inversion or flattening in the lateral or inferolateral leads. The ECG, nevertheless, can often appear normal. Additionally, ALVC often exhibits ventricular arrhythmias with a right bundle branch block (RBBB) morphology. Furthermore, individuals with ALVC may have normal or mildly depressed LV systolic function along with little to no dilatation of the LV. Contrast-enhanced cardiac magnetic resonance (CMR) imaging demonstrates a large amount of myocardial fibrosis, evidenced by late gadolinium enhancement (LGE), mainly involving the subepicardial layers of the inferior and inferolateral regions in a non-ischemic pattern [2, 3, 5]. The 2019 diagnostic criteria for ALVC placed significant emphasis on assessing electrocardiographic, structural, and functional changes associated with LV involvement. However, genetic testing utilizing advanced techniques such as next-generation sequencing (NGS) is required for diagnostic confirmation [5–8]. Most mutations that result in ACM affect genes that encode structural proteins involved in the construction of intercellular junctions, especially desmosomal proteins. These include plakophilin-2 (*PKP2*), desmoplakin (*DSP*), desmoglein-2 (*DSG2*), and desmocolin-2 (*DSC2*). Studies indicate that the most frequent gene deficiencies causing left-sided ACM are mutations in the *DSP*, phospholamban (*PLN*), and filamin C (*FLNC*) genes [9]. Approximately 2–12% of these patients have DSP mutations [10]. The *DSP* gene, located on chromosome 6p24.3, codes for desmoplakin, which links intermediate filament proteins to desmosomal plaques and is necessary for functional desmosomes. Desmoplakin mutations result in a loss of function, leading to intercellular adhesion failure. These mutations have been associated with fibrosis and inflammatory infiltrates in the ventricular myocardium, as observed in histological examinations [11–13]. In this investigation, we identified a novel heterozygous mutation in the *DSP* gene utilizing NGS technology. The mutation was found in an Iranian family with cardiomyopathy and sudden cardiac death, suggesting that it may be responsible for the genetic basis of this condition.

## Method

### Clinical features

A three-generation Iranian family with SCD, myocardial infarction, and heart failure was recruited in this study. The proband, a 43-year-old woman with a 2-year history of several presyncope episodes, presented with mild chest pain, mild dyspnea, and early exercise intolerance. Upon thorough cardiovascular examination, severe systolic dysfunction with mild LV enlargement was demonstrated. A family investigation found that the proband's father and brother had SCD at the age of 70 and 26, respectively. Furthermore, her sister had been diagnosed with heart failure (HF) several years earlier and had undergone pacemaker implantation at the age of 31. CMR was performed for all available family members. The proband was referred to the Cardiogenetics Research Center at Rajaie Cardiovascular Medical and Research Center, Iran University of Medical Sciences, Tehran, Iran, to conduct genetic testing and further investigate the underlying genetic factors contributing to the clinical manifestations.

### Cardiac magnetic resonance imaging (CMR)

CMR was performed using the 1.5Tesla Magnetom Sola, Siemens Healthcare, Erlangen, Germany. Steady-state free precession (SSFP) functional imaging with breath-holding was performed in the four-, two-, and three-chamber (long-axis views) and short-axis stacks to assess left and right ventricular function. Right ventricular outflow and inflow-outflow views were also acquired. Short Tau inversion recovery (STIR) sequence (breath-hold) in long- (four-, two-, and three-chamber) and short-axis was taken to assess inflammation. Gadolinium injection was done with Gadoterate meglumine (gadolinium-DOTA, Dotarem, Guerbet S.A., Paris, France) 0.15 mmol/kg. The magnitude and phase-sensitive inversion recovery reconstructions of early and late gadolinium images were taken in the short-axis stack, four-, two-, and three-chamber views.

### Whole-exome sequencing and variant confirmation

Blood samples were gathered from the patient and family members. Genomic DNA was isolated from peripheral blood leukocytes using the Roche kit (DNSol Mini kit of Roche: lot No. 500021091210050). Then, the quality of the extracted DNA was checked by measuring the optical density in the Nanodrop2000 device (Thermo Scientific). 2 μg of the proband DNA was separated for NGS, which was performed on the Illumina HiSeq 6000 sequencer (Macrogen). The raw data was analyzed by Rajaie Cardiovascular, Medical, and Research Center, Tehran, Iran. After quality control checks on the raw sequence data using FastQC, the reads were aligned against the reference genome GRCh37 (hg19) using Burrows Wheeler Aligner software. The SAM files were trimmed using the Picard tools, and then the resulting BAM files were realigned using the GATK pipeline. After that, the GATK pipeline haplotype caller module was used to call the variants, and the identified variants were annotated using the ANNOVAR tool. Filtering of the variants according to a standard analysis pipeline was performed by public variable frequency databases, such as 1000 Genomes, Exome Sequencing Project and GnomAD Browser, ExAC 12 Browser, and Iranome (http://www.iranome.ir). Then the variants were prioritized based on gene ontology, clinical significance, and bioinformatic prediction scores. The Combined Annotation Dependent Depletion (CADD) algorithm and MutationTaster (http://www.mutationtaster.org/) were used for predicting the functional trace of mutations. The pathogenicity of the identified variants was also assessed using the guidelines of the American College of Medical Genetics (ACMG) [14].

### Polymerase chain reaction (PCR), primer design, and Sanger sequencing

For mutation confirmation, family member evaluation, and segregation analysis, we designed specific primers for polymerase chain reaction (PCR) amplification of candidate causative mutations identified in the patient. For designing PCR primers, we used the program GeneRunner. PCR reactions were performed as follows: Denaturation (95 °C for 30 s), annealing (60 °C for 30 s), and extension (72 °C for 35 s). The PCR products were performed using the ABI Genetic Analyzer 3500XL, and the data were analyzed using the BioEdit program (v.7.0.5.3).

## Result

### Clinical investigations

**Fig. 1:**
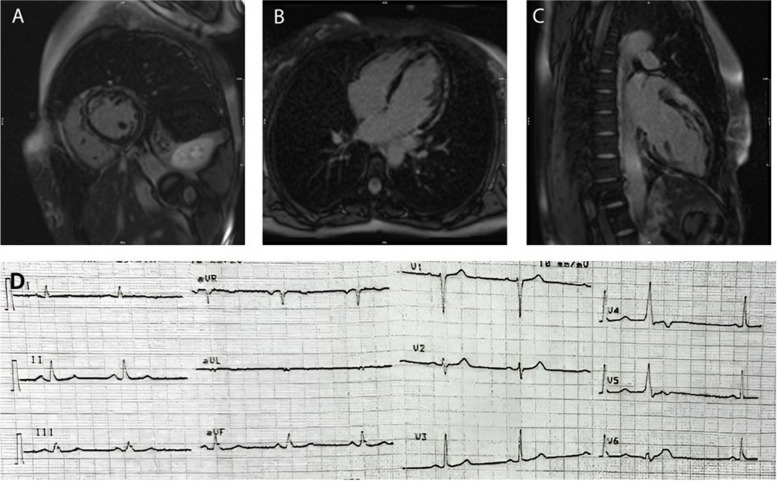
The clinical information of the patient. **A**-**C** The CMR late enhancement images at the mid-level short-axis, four-chamber and two chambers view, respectively, show circumferential patchy subepicardial enhancement in the basal to apical segments of the LV myocardium. **D** the Electrocardiogram (ECG) of the proband revealed a normal sinus rhythm, but with T wave inversion in the V4 and V5 leads

The CMR showed mildly increased left ventricle size with left ventricular end-diastolic volume indexed to body surface area (LVEDVI) of 103 ml/m^2^ and a mildly reduced left ventricular ejection fraction (LVEF) of 49%. Regional wall motion abnormality was evident as hypokinesia in the mid-lateral, mid-septal, and mid-anterior walls (Supplementary video 1). The right ventricle size was normal, and ejection fraction with end-diastolic volume indexed to body surface area (RVEDVI) of 80 ml/m^2^ and RVEF of 66%. No regional wall motion abnormality was evident in the right ventricle. (Supplementary video 2). The STIR sequences showed no inflammation or oedema. Late gadolinium enhancement sequences showed circumferential patchy subepicardial enhancement in the basal to apical segments of the left ventricular myocardium (Fig. 1 A-C). Considering the familial history of heart failure and SCD, the CMR features of left ventricular dysfunction and the circumferential subepicardial pattern of myocardial fibrosis were compatible with the phenotype of ALVC. In addition, the 12-lead ECG revealed a normal sinus rhythm, but with T wave inversion observed in the V4 and V5 leads (Fig. 1 D). The proband sister also showed almost the same CMR and ECG findings. The clinical assessments conducted on other available individuals within the pedigree indicated no abnormal findings.

### Genetic investigations (Results of Genetic Testing)

**Fig. 2:**
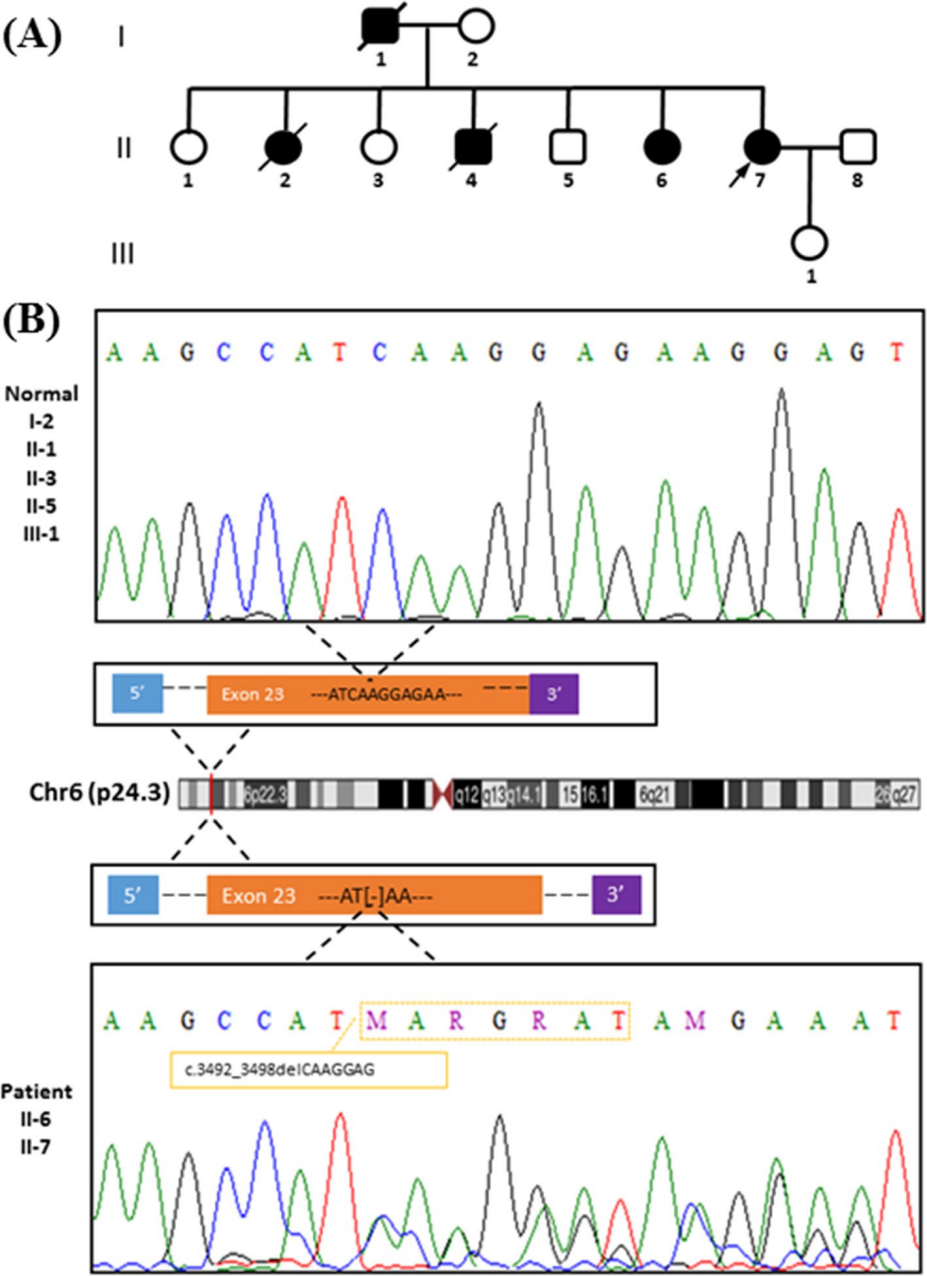
The image illustrates family pedigree and genetic analysis of a *DSP* c.3492_3498delCAAGGAG variant. A The pedigree of an Iranian family is shown herein. B The genotype of a novel pathogenic variant c.3492_3498delCAAGGAG:p. K1165Rfs*8 located in the region of exon 23 were detected as heterozygous in the affected proband (II-7) and in his sister (II-6). The other individuals had a normal sequence

After exome sequencing, a novel likely pathogenic heterozygous variant was identified in the proband (Fig. 2A: II-7), *DSP*(NM_004415.4), c.3492_3498del, p.K1165Rfs*8. This variant was confirmed by Sanger sequencing and segregated in other affected members of the family (Fig. 2B). The healthy individuals of the pedigree have a normal sequence in this position. The c.3492_3498del variant was predicted to be disease-causing by Mutation Taster. The CADD phred of this variant was 33.

We also investigated other DSP gene variants associated with ALVC and studied their genetic and clinical characteristics. To accomplish this, we conducted a literature search on PubMed and Google Scholar, using the search terms 'arrhythmogenic left ventricular cardiomyopathy' or 'arrhythmogenic cardiomyopathy' and 'DSP.' The results of this search are summarized in Table 1.

**Table 1:** The reported *DSP* variant associated with ALVC and their related phenotypes

No	Nucleotide change	Amino acid change	dbSNP	CADD	Mutation Taster	ECG Abnormality (n)	LV Dysfunction (n)	LV Dilatation (n)	LV Epicardial LGE (n)	Arrythmia (n)	ICD (n)	SCD History in Family	Other Organ Involvement	Ref
1	c.7113_7119delGATCGCA	p.I2372Pfs*17	-	35	D	0/1	1/1 (Mild)	0/1	1/1	0/1	0/1	-	-	[24]
2	c.2521_2522delCA	p.Q841Dfs*9	-	33	D	2/3	1/2 (Mild)	0/2	3/3	1/2 (NSVT)	0/3	-	-	[25]
3	c.2034insA	p.T586fs*594	-	37	D	8/11	10/11 (Mild)	8/11 (Mild to moderate)	4/4	11/11 (PVC, SVT, LBBB, RBBB)	3/11	+	N/A	[26]
4	c.5596A > T	p.R1866X	rs1199138047	38	D	3/4	5/10 (Mild)	1/10 (Mild)	7/9	4/10 (RBBB, LBBB,SVT)	6/10	+	-	[17, 27]
5	c.448C > T	p.R150*	-	40	D	2/3	0/2	0/2	2/2	1/1 (PVC and RBBB)	0/3	+	-	[28]
6	c.3526deLG	p.V1176fs*20	rs727505271	27.4	D	0/1	1/1 (Severe)	0/1	1/1	1/1 (NSVT)	0/1	N/A	N/A	[28]
7	c.2920deLA	p.T974fs*3	rs727505260	33	D	0/1	0/1	1/1 (Mild)	1/1	1/1	0/1	N/A	N/A	[28]
8	c.3434deLC	p.A1145fs*14	rs1581815603	23.6	D	0/1	N/A	N/A	N/A	1/1	0/1	N/A	N/A	[28]
9	c.3924delG	p.H1309Tfs*40	-	32	D	2/3	0/3	0/3	2/2	2/3 (PVC)	N/A	+	N/A	[29]
10	c.1865delT	p.L622Rfs*14	-	33	D	1/1	1/1 (Moderate)	1/1 (Mild)	1/1	1/1 (NSVT)	N/A	+	N/A	[29]
11	c.1396C > T	p.L466F	-	25.8	D	1/2	1/1 (Moderate)	2/2 (Mild)	2/2	1/2 (NSVT)	N/A	-	N/A	[29]
12	c.2610delA	p.I870Mfs*19	-	34	D	1/1	1/1 (Severe)	1/1 (Mild)	1/1	1/1 (PVC)	N/A	+	N/A	[29]
13	c.3211C > T	p.Q1071*	-	40	D	1/1	0/1	0/1	1/1	1/1 (NSVT)	N/A	+	N/A	[29]
14	c.5212C > T	p.R1738*	rs794728124	36	D	1/1	1/1 (Mild)	0/1	1/1	1/1 (NSVT and RBBB)	1/1	-	-	[30]
15	c.4372C > T	p.R1458*	rs28763965	37	D	2/2	1/2 (Mild)	1/2 (Mild)	2/2	1/2 (NSVT)	0/3	-	-	[31]
16	c.3533T > G	p.L1178R	-	24.2	D	0/1	0/1	0/1	1/1	0/1	1/1	+	-	[32]
17	c.4788delA	p.E1596f + 5*	-	34	D	0/2	N/A	N/A	N/A	N/A	1/3	+	-	[33]
18	c.6310delA	p. T2104Qfs*12	rs730880092	34	D	10/12	11/12 (Mild to severe)	9/12 (Mild to severe)	4/4	5/12 (VT, RBB, LBBB)	6/12	+	-	[12]
19	c.1080G > A	p.W360*	-	39	D	1/2	1/2 (Mild)	1/2 (Mild)	N/A	1/2 (NSVT)	0/2	+	-	[34]
20	c.5428C > T	p.Q1810*	rs397516946	40	D	3/3	3/3 (Mild)	0/3	3/3	0/3	1/3	-	-	[35]
21	c.3889A > T	p.R1297W	rs1356287373	37	D	1/2	0/2	0/2	2/2	0/2	0/2	N/A	-	[36]
22	c.1067C > A	p. T356K	rs780626687	21	D	N/A	0/2	0/2	3/3	N/A	3/3	+	-	[37]
23	c.2811_2812dupAT	p.S938Yfs*	-	27	D	2/3	1/3 (Moderate)	0/3	1/1	1/3 (SVT)	1/3	-	Cutaneous phenotype	[38]
24	c.4789G > T	p. Q1597*	rs397516943	43	D	1/1	1/1 (Mild)	1/1 (Mild)	2/2	1/2 (SVT)	1/2	-	-	[39]
25	c.478 C > T	p. R160*	rs397516943	35	D	1/1	1/1 (Severe)	1/1 (Mild)	1/1	1/1 (SVT and RBBB)	1/1	-	Cutaneous phenotype	[18]
26	c.1267-2A > G	-	-	-	-	1/1	0/1	0/1	1/1	1/1 (NSVT)	0/1	-	-	[40]
27	c.218_219insAT	p.L74C	-	29	D	1/1	1/1 severe	1/1 severe	1/1	1/1 (SVT)	1/1	-	-	[40]
28	c.3082_3084 + 13del	-	-	-	-	1/1	1/1 (Mild)	0/1	1/1	0/1	N/A	-	-	[41]
29	c.3403C > T	p.Q1135*	-	37	D	1/1	0/1	0/1	1/1	0/1	N/A	-	-	[41]
30	c.2588T > C	p.L863P	rs1357944906	29.1	D	0/1	1/1 (Mild)	0/1	1/1	0/1	N/A	+	-	[41]
31	c.5028_5031delACAA	p.Q1677Lfs*3	-	33	D	1/1	1/1 (Mild)	0/1	1/1	0/1	N/A	+	-	[41]
32	c.1904-2A > G	-	-	-	-	1/1	1/1 (Mild)	1/1 (Mild)	1/1	1/1 (NSVT)	1/1	-	-	[42]
33	c.5851C > T	p.R1951*		37	D	1/1	1/1 (Mild)	1/1 (Mild)	1/1	1/1 (SVT)	1/1	+	-	[43]
34	c.8586delC	p. S2863Hfs*20	-	31	D	3/4	0/4	2/4 (Mild)	1/1	2/3 (SVT and PVC)	1/4	+	-	[44]
35	c.4513G > A	p.A1505T	rs1488770025	13.03	P	0/1	1/1 (Mild)	N/A	N/A	0/1	N/A	-	N/A	[45]
36	c.6697_6698delGT	p.V2233Qfs*2	-	33	D	0/1	1/1 (Severe)	1/1	1/1	1/1 (PVC)	1/1	-	-	[46]

*CADD* Combined Annotation-Dependent Depletion, *dbSNP* Database for Single Nucleotide Polymorphisms and Other Classes of Minor Genetic Variation, *D* Disease-causing, *ICD* Implantable Cardioverter Defibrillator, *LBBB* Left Bundle Branch Block, *LGE* Late Gadolinium Enhancement, *LV* Left Ventricle, *N/A* Not Applicable, *NSVT* Non-Sustained Ventricular Tachycardia, *PVC* Premature Ventricular Contraction, *P* Polymorphism, *RBBB* Right Bundle Branch Block, *SCD* Sudden Cardiac Death, *SVT* Sustained Ventricular Tachycardia

## Discussion

**Fig. 3:**
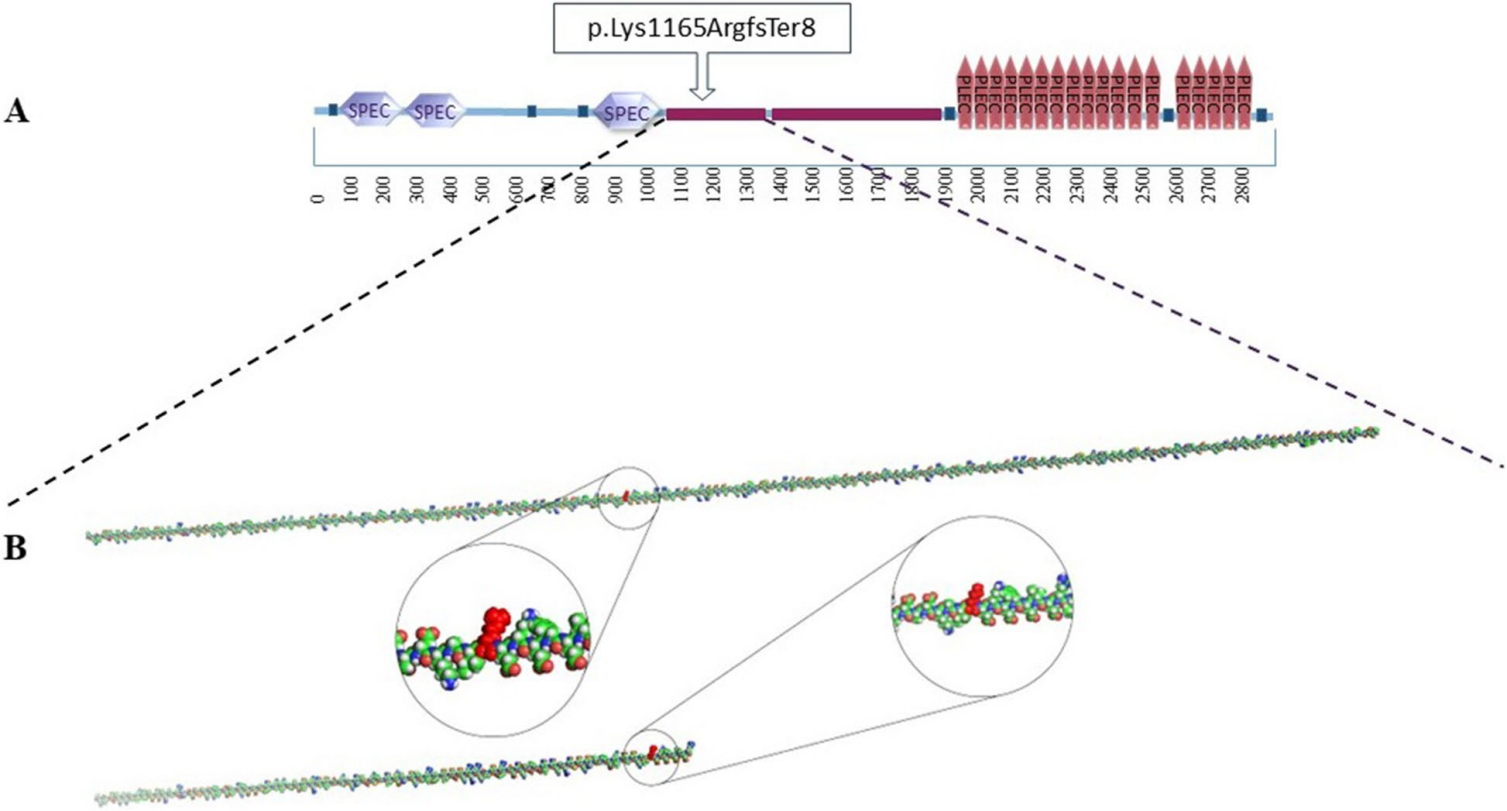
The structure of Desmoplakin protein. A The secondary structure of the DSP. B The 3-D structure of the wild and mutant DSP protein

The newly updated Padua criteria for diagnosing ALVC incorporate functional, structural, electrocardiographic abnormalities, and family genetic background. Table 2 provides a summarized overview of this diagnostic criteria [2, 15]. Identifying a pathogenic mutation in genes associated with ALVC is the most specific finding that establishes a link between the ALVC genotype and phenotype features. Therefore, familial background of first-degree relatives is considered a major criterion. According to Padua criteria, the occurrence of premature SCD in first-degree relatives under the age of 35 raises diagnostic suspicion to ACM. A recent autopsy study showed that 87% of ACM-related SCD patients have predominant LV involvement. It seems genetic testing in such cases can identify family members who are genetically affected in preclinical phase of disease. The fact that pathogenic variants in cardiomyopathy genes can potentially lead to SCD without obvious structural abnormalities highlighted the importance of early diagnosis of this condition [2, 15, 16]. ALVC is commonly associated with genes involved in encoding desmosomal proteins, namely Desmoplakin (*DSP*), Filamin C (*FLNC*), Phospholamban (*PLN*), and Desmin (*DES*). Among these genes, DSP mutations have emerged as the most frequently observed disease-associated mutations in ALVC patients. Both missense and non-missense mutations in the DSP gene pose a significant risk for arrhythmias and SCD. Notably, non-missense mutations are closely associated with left-dominant forms of the disease. In our study, we identified a novel autosomal dominant frameshift mutation (*DSP*, c.3492_3498del, p.K1165Rfs*8) that leads to a truncated desmoplakin protein with a premature stop codon at position 1165. This mutation contributes to the development of a left-dominant cardiomyopathy. Desmoplakin is a large protein consisting of three distinct domains. These domains include an N-terminal plakoglobin/plakophilin binding domain, a central coiled-coil rod dimerization domain, and a C-terminal intermediate filament binding domain (Fig. 3). Desmoplakin holds significant importance as a member of the protein family responsible for cell–cell adhesion within desmosomes, which play a crucial role in maintaining the integrity of both cardiomyocytes and epithelial tissues. Truncation variants of desmoplakin result in loss of function, leading to a failure in intercellular adhesion. Consequently, this disruption triggers cardiomyocyte death, leading to inflammation and fibrosis [7, 17–22]. Mutations in desmosomal genes not only affect cell adhesion and structural integrity but also disrupt intracellular signaling processes. These mutations can interfere with signaling pathways involved in communication between the nucleus and desmosomes, as well as impact the function of gap-junctional proteins and ion channels. Recent research has highlighted the regulatory role of specific desmosomal proteins, such as plakophilin 2 and desmoplakin, in the TGFβ signaling pathway. When PKP2 or DSP is lost or impaired, it leads to heightened TGFβ signaling, resulting in the expression of genes associated with fibrosis and the accumulation of collagen. Additionally, the suppression of desmoplakin has been found to inhibit the Wnt–β-catenin signaling pathway, which in turn triggers adipogenesis and the proliferation of adipocytes. These dysregulated signaling pathways contribute to the development of fibrosis, providing a biological basis for understanding the underlying mechanisms of fibrotic diseases [19, 23]. This study explores the potential impact of *DSP*, c.3492_3498del, p.K1165Rfs*8 variant on the phenotype of ALVC. However, it has certain limitations. Firstly, the DNA samples of the individuals who experienced SCD were not accessible, which hindered further investigation into genotype–phenotype correlations. Secondly, while our bioinformatics analysis indicated the pathogenic nature of the variant, conducting functional evaluations could have provided additional confirmation of its pathogenicity.

**Table 2:** Newly updated Padua criteria for diagnosing ALVC

Criteria Category	Major Criteria	Minor Criteria
Functional abnormality		• LV systolic dysfunction • Regional LV Wall Hypokinesia or Akinesia
Structural myocardial abnormalities	• LV Subepicardial or Midmyocardial LGE	
Electrocardiographic abnormalities		• Inverted T Waves in Precordial Leads V4-V6 (in absence of LBBB) • Low QRS voltages • RBBB Morphology Ventricular Arrhythmias
Familial Inheritance and Molecular Genetics	• Confirmed ACM in First-Degree Relative • Identification of Pathogenic or Likely Pathogenetic ACM Mutation in Patient	• History of ACM in First-Degree Relative with Indeterminate Diagnostic Criteria • Premature Sudden Death (< 35 years of age) Suspected to be Due to ACM in First-Degree Relative • Pathological Confirmation or Diagnostic Criteria Confirmation of ACM in Second-Degree Relative

*LV* Left ventricle, *LGE* Late gadolinium enhancement, *LBBB* Left bundle branch block, *RBBB* Right bundle branch block, *ACM* Arrhythmogenic cardiomyopathy

## Conclusion

The *DSP* gene is widely recognized as a significant contributor to ALVC. This study introduces a novel likely pathogenic variant, c.3492_3498del, p. K1165Rfs*8, in the *DSP* gene, which has been identified as the cause of ALVC in an Iranian family. Our investigation underscores the importance of genetic testing, specifically WES, for individuals suspected of ALVC and have a family history of SCD.

### Supplementary Information

**Additional file 1: Supplementary video 1.** The four-chamber cine image shows left ventricular dysfunction.

**Additional file 2: Supplementary video 2.** The RV inflow outflow cine image shows no regional wall motion abnormality.

## Data Availability

The datasets generated and/or analyzed during the current study are available in the ClinVar repository [https://www.ncbi.nlm.nih.gov/clinvar/variation/2506005/]. The accession number of the variant in ClinVar is as follows: NM_004415.4 (DSP): c.3492_3498del (p.Lys1165ArgfsTer8): VCV002506005.1
